# A new model for compressor surge and stall control

**DOI:** 10.1038/s41598-024-55816-w

**Published:** 2024-03-04

**Authors:** M. J. Shahriyari, A. Firouzabadi, H. Khaleghi, S. M. Esmailifar

**Affiliations:** Department of Aerospace Engineering, Amirkabir University of Technology, Tehran, 15875 4413 Iran

**Keywords:** Power stations, Power stations, Mechanical engineering

## Abstract

This paper compares the bifurcations and closed-loop performances of two compressor models, Moore-Greitzer (MG) and a developed model based on MG (Shahriyari Khaleghi, SK). First, both models are linearized about two equilibrium points (pure surge and fully-developed rotating stall), and the perturbed state-space dynamics and input matrices are obtained. The compressor unstable regions are then identified using an eigenvalue and global bifurcation analysis. Furthermore, optimal LQR controllers are designed, and the performances of closed-loop systems are compared. The LQRs are designed to control the compression system near the peak pressure rise by suppressing surge or stall. Results reveal that if the initial operating point is in the positive slope region of the compressor characteristic and the initial amplitude of the disturbances is small, the LQR controller can stabilize the compressor in both models. However, when the disturbances are intensive, the two models respond differently: although the SK model damps a fair range of disturbances and predicts instability for excessively powerful disturbances, the MG model always damps them, even when extremely intense. Without a controller in the MG model, initial disturbances (even very large) can never grow and are always damped in the compressor’s negative slope region (obviously, the same applies to the controller). However, pending the amplitude of the disturbances (in the absence of a controller), the disturbances in the SK model may be damped or grow. The SK model can successfully control the instabilities if the disturbances are small. Nonetheless, the controller fails to dampen the instabilities for extreme disturbances, which is consistent with reality.

## Introduction

The main limitation in the operation of aero compressors is the onset of two aerodynamic instabilities, the so-called surge and rotating stall, and therefore, it is always desired to postpone the occurrence of these instabilities by control methods. Surge is defined as large amplitude oscillations of the annulus flow over the whole compression system, whereas rotating stall is a limited disturbance that may be limited to one or some compressor stages. There are two patterns for rotating stall inception: modal (long-length scale disturbances) and spike (short-length scale disturbances)^1,2^. Modal-type stall inception was first proposed by^3,4^ before being experimentally observed and studied by some researchers^5–7^. This stall pattern has been observed in low-speed as well as high-speed compressors^5,6^ and includes the gradual growth of long-length scale disturbances before the formation of stall cells and, therefore, can be detected by employing suitable sensors. In contrast, in spike-type stall inception, the stalling disturbances rapidly lead to the formation of stall cells^8,9^. Also, models similar to Moore^3^ and Moore and Greitzer^4^ were developed by many researchers due to their importance in identifying parameters affecting instability^10–13^.

The currently operating strategy used in gas turbine engines is the so-called “surge avoidance”, which is a passive approach and maintains stable operation at a sufficient margin from the surge line. In this strategy, the compressor cannot work near the surge line, where the pressure rise is maximum^14^. Epstein et al^14^. proposed the concept of intelligent engines, in which the compressor is allowed to operate close to the surge line. This leads to an active control strategy, the so-called stall detection and control, in which control devices shall detect and remove any emerging stall disturbances^15,16^. The model developed by Moore-Greitzer^4^ can model surge and stall disturbances and is suitable for compressor control studies. Since then, many researchers have studied MG mode to control surge and rotating stall. Liaw and Abed^17^ developed a nonlinear controller based on the nonlinear characteristics of the MG model^4^. They applied bifurcation theory to actively control compressor stall inception by eliminating the unwanted jump and hysteresis behavior of the compression system. Most compressor control research in the late 20th and early 21st centuries focused on investigating the qualitative behavior and bifurcation analysis of the MG model to investigate the impact of the model parameters on the instabilities^17–21^. Most of these studies have developed stall/surge controllers and addressed the significance of the throttle gain as an MG model parameter for bifurcation analysis. During the last two decades, classical and advanced algorithms such as linear feedback stabilization^22,23^, nonlinear feedback control^24,25^, fuzzy systems^26^, sliding mode control^27^, model predictive control^28,29^ and passivity control^21,30^ have been employed to control the compressor system.

Among classical linear control methods such as PID and pole placement, LQR controllers perform quite well. The LQR design method is an optimal full-state feedback control that outperforms the PID controller, showing better settling time, rise time, and overshoot response^31,32^.

Table 1 reviews the most notable efforts in the active control design of surge and rotating stalls for axial compressor systems over the last two decades.

**Table 1:** Some of the most notable recent efforts in active control design for axial compressor systems.

Year	Author	Model	Control approach	Actuator	Controlled phenomenon
1998	Krstic et al.^33^	MG	Back-stepping	Throttle	Surge/Stall
1999	Gravdahl^30^	Ext. MG	Passivity-Based	CCV	Surge/Stall
2001	Liaw and Huang^34^	MG	FOSMC	CCV	Surge/Stall
2002	Liaw et al.^35^	MG	Lyapunov	Throttle + CCV	Surge
2003	Ananthkrish et al.^36^	MG	Bifurcation	Throttle	Surge/Stall
2007	Wang and Murray^25^	MG	Bifurcation	Throttle	Surge/Stall
2010	Vepa^37^	Ext. MG	Nonlinear	Pressure	Stall
2011	Moghaddam and Madani^27^	MG	NFuzzy + SMC	Throttle	Surge/Stall
2013	Chen and Xu^24^	MG	Nonlinear	Throttle	Surge/Stall
2018	Sari et al.^21^	Ext. MG	Passivity-Based	Throttle + CCV	Surge/Stall
2020	MFW Chowdhury, MP Schoen, J Li^38^	MG	Fuzzy Logic	Throttle	Surge/Stall
2020	Å Neverlien, S Moe, JT Gravdahl^39^	MG	Lyapunov Neural Networks	Throttle + CCV	Surge
2023	Ning Su and Yong Wang^40^	MG	quadratic feedback	Throttle	Surge/Stall

Shahriyari et al^41^. developed a model based on Moore-Greitzer equations by adding the second-order derivative of the flow coefficient to the hysteresis of the compressor pressure rise function. This model has some advantages compared to the basic Moore-Greitzer model. It can model the transient behavior of the stall cell. Furthermore, the slope of the compressor characteristic curve is included in the governing equations, which enables stall inception when the initial operating point is on the negative slope portion of the compressor characteristic curve (which might occur in real applications^8^, but is not modeled in the basic Moore-Greitzrer, MG). The developed model also includes the rate of throttling, which is not included in Moore-Greitzer equations (Shahriyari et al^41^.). Considering the new capabilities of the SK model, and that it has not been studied by researchers yet, it is essential to investigate its dynamic behavior and compare it to the MG model, before more detailed linear and nonlinear investigations being performed by researchers.

This study aims to examine the effect of refining the accuracy of the Shahriyari et al^41^. (SK) model on the controlled compressor’s closed-loop performance, comparing it to the closed-loop performance of the conventional MG model. Additionally, bifurcation analysis is performed on the upgraded model, and the results are contrasted with those of the MG model. Equilibrium points, including pure surge and fully developed rotating stall, are determined first, followed by the development of linearized models around these points. Bifurcation analysis then investigates the behavior and stability of these equilibrium points as a function of throttle valve position. Moreover, an LQR controller is designed based on state-space linear models to suppress rotating stall and surge limit cycles by throttle valve manipulation. Finally, the control behaviors of the two systems are compared in detail.

Given the enhanced capabilities of the SK model^41^ compared to the conventional MG model, this study’s significance lies in its exploration of bifurcation behavior and closed-loop performance of the improved model. The outcomes of this study provide valuable insights for researchers to control dynamic modes that may not be adequately captured by the basic MG model, such as initial operating points beyond peak pressure rise or under varying throttle rates. However, a significant limitation of this study is the utilization of a linear LQR controller, which may not adequately handle the compressor under severe disturbances.

## Compressor instability equations

**Figure 1:**
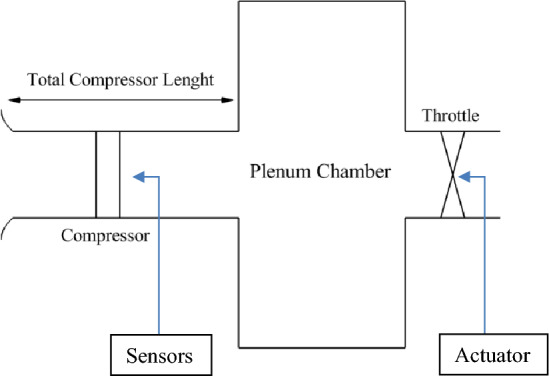
Compression system.

**Figure 2:**
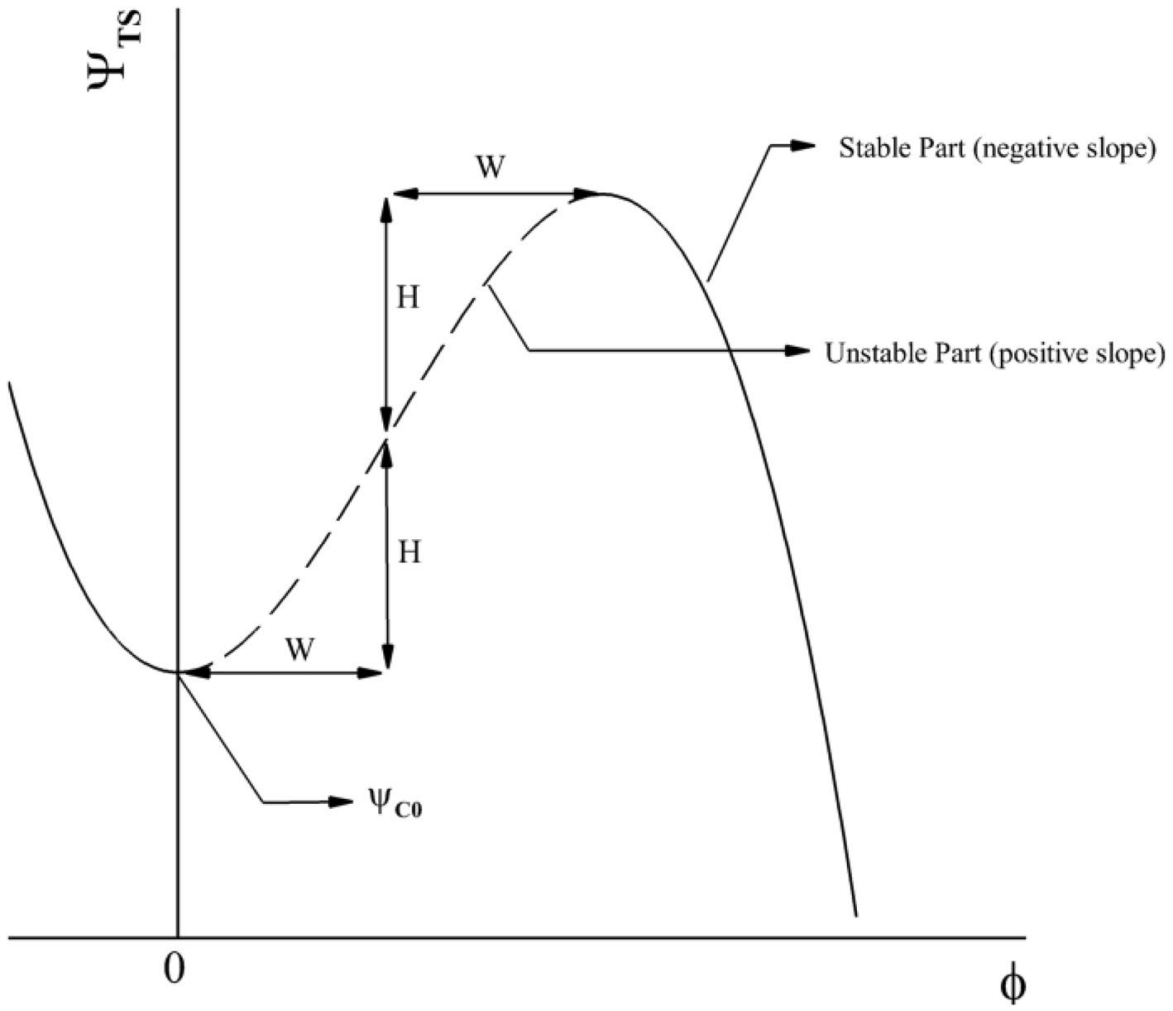
Compressor pressure rise characteristic.

Figure 1 illustrates the compression system used in this study. The flow is assumed to be two-dimensional and inviscid throughout the system. The axial and circumferential coordinates are represented by η (axial distance divided by fan mean radius) and θ (wheel angle), respectively. Furthermore, the compressor axisymmetric characteristic is given in Fig. 2 and Eq. 1. The parameters ψ_c0_ (Compressor pressure rise coefficient at zero mass flow rate), H (semi-height of cubic axisymmetric characteristic), and W (semi-width of cubic characteristic) in this equation are equivalent to 0.2, 0.18 and 0.25, respectively.

1 $$\psi_{c} \left( \phi \right) = \psi_{C0} + H\left[ {1 + \frac{3}{2}\left( {\frac{\Phi }{W} - 1} \right) - \frac{1}{2}\left( {\frac{\Phi }{W} - 1} \right)^{3} } \right]$$

where ψ_c_ is the axisymmetric pressure rise coefficient and Φ is the annulus averaged axial flow coefficient of the compressor.

2 $$\frac{d\Psi }{{d\xi }} = \frac{1}{{4l_{C} B^{2} }}\left[ {\Phi - \gamma_{T} \sqrt \Psi } \right]$$

3 $$\frac{d\Phi }{{d\xi }} = \left[ { - \frac{{\Psi - \psi_{C0} }}{H} + 1 + \frac{3}{2}\left( {\frac{\Phi }{W} - 1} \right)\left( {1 - \frac{1}{2}A^{2} } \right) - \frac{1}{2}\left( {\frac{\Phi }{W} - 1} \right)^{3} } \right]\frac{H}{{l_{c} }}$$

4 $$\frac{dA}{{d\xi }} = \,\frac{3}{2}\frac{AH}{W}\left( {m + \frac{1}{a}} \right)^{ - 1} \left[ {1 - \left( {\frac{\Phi }{W} - 1} \right)^{2} - \frac{1}{4}A^{2} } \right]$$

in which ξ is the non-dimensional time (U t/R), *A* is the amplitude of the disturbances, l_c_ is the equivalent compressor length, and *m* and *a* are the external and internal compressor lag respectively. In addition, ${\gamma }_{T}$ represents the position of throttle valve that is acted as control signal. ${\gamma }_{T}$ can be between 0 (closed mode) and 1 (fully open). In this paper: m = 1.75, 1/a = 3.5, and l_c_ = 8.

The governing equations of the model proposed by Shahriyari et al^41^. are given in Eqs. 5–8. According to Shahriyari et al^41^., the proposed model has the advantage of including the rate of throttling and the slope of the compressor characteristic. In addition, the transient behavior of the stall cell can be predicted by this model, and instabilities can also be simulated if it starts from the negative slope part of the compressor characteristic, which is not possible in the basic Moore-Greitzer model.

5 $$\frac{d\Psi }{{d\xi }} = \frac{1}{{4l_{C} B^{2} }}\left[ {\Phi - \gamma_{T} \sqrt \Psi } \right]$$

6 $$\mp \frac{{2\psi^{\prime}_{c} }}{Z}\frac{{d^{2} \Phi }}{{d\xi^{2} }} + l_{c} \frac{d\Phi }{{d\xi }} = \left[ { - \frac{{\Psi - \psi_{C0} }}{H} + 1 + \frac{3}{2}\left( {\frac{\Phi }{W} - 1} \right)\left( {1 - \frac{1}{2}A^{2} } \right)\,\, - \frac{1}{2}\left( {\frac{\Phi }{W} - 1} \right)^{3} } \right]H$$

7 $$\begin{gathered} \mp \frac{{2\psi^{\prime}_{c} }}{Z}\frac{{d^{2} A}}{{d\xi^{2} }} + \left( {m + \frac{1}{a}} \right)\frac{dA}{{d\xi }} + \left( { \pm \frac{{2\psi^{\prime}_{c} }}{Z}\left( {\frac{dr}{{d\xi }}} \right)^{2} \mp \frac{{2\psi^{\prime}_{c} }}{Z}\frac{dr}{{d\xi }} \pm \frac{{\psi^{\prime}_{c} }}{Z}} \right)A = \hfill \\ \,\,\,\,\,\,\,\,\,\,\,\,\,\,\,\,\,\,\,\,\,\,\,\,\,\,\,\,\,\,\,\,\,\,\,\,\,\,\,\,\,\,\frac{3}{2}\frac{AH}{W}\left[ {1 - \left( {\frac{\Phi }{W} - 1} \right)^{2} - \frac{1}{4}A^{2} } \right] \hfill \\ \end{gathered}$$

8 $$\pm \frac{{2\psi^{\prime}_{c} }}{Z}A\frac{{d^{2} r}}{{d\xi^{2} }} + \left( { \pm \frac{{4\psi^{\prime}_{c} }}{Z}\frac{dA}{{d\xi }} - mA - \frac{1}{a}A} \right)\frac{dr}{{d\xi }} + \left( {\frac{1}{2a}A \mp \frac{{2\psi^{\prime}_{c} }}{Z}\frac{dA}{{d\xi }}} \right) = 0$$

where Z is defined as follows:

9 $$Z = \left( {\frac{\frac{1}{2}}{1 + am}} \right)^{2} + \left( {\frac{\frac{1}{2}}{1 + am} - 1} \right)^{2}$$

### State-space model

Nonlinear dynamic models are typically represented by first-order time differential equations in state space as $\dot{{\varvec{x}}}={\varvec{f}}({\varvec{x}},{\varvec{u}})$, where ***x*** is state vector and ${\varvec{u}}$ is control input. Therefore, to model the dynamic system, it is necessary to first define the state variables (components of state vector ***x***). Considering the states of the Moore-Greitzer model as ${x}_{1}=\Psi$, ${x}_{2}=\Phi$, ${x}_{3}=A$, and the control input as ${u}_{1}={\gamma }_{t}$ (represents the throttle valve position, ${\gamma }_{T}\epsilon \left[\mathrm{0,1}\right]$ from closed mode to fully open), Eqs. 10–12 represent the state space of the Moore-Greitzer model.

10 $$\dot{x}_{1} = \frac{1}{{4l_{C} B^{2} }}\left[ {x_{2} - u_{1} \sqrt {x_{1} } } \right]$$

11 $$\dot{x}_{2} = \left[ { - \frac{{x_{1} - x_{1C0} }}{H} + 1 + \frac{3}{2}\left( {\frac{{x_{2} }}{W} - 1} \right)\left( {1 - \frac{1}{2}x_{3}^{2} } \right)\, - \frac{1}{2}\left( {\frac{{x_{2} }}{W} - 1} \right)^{3} } \right]\frac{H}{{l_{c} }}$$

12 $$\dot{x}_{3} = \,\frac{3}{2}\frac{H}{W}\left( {m + \frac{1}{a}} \right)^{ - 1} \left[ {1 - \left( {\frac{{x_{2} }}{W} - 1} \right)^{2} - \frac{1}{4}x_{3}^{2} } \right]x_{3}$$

Considering the states of the modified model as ${x}_{1}=\Psi$, ${x}_{2}=\Phi$, ${x}_{3}=d\Phi /d\xi$, ${x}_{4}=A$, ${x}_{5}=d{\text{A}}/d\xi$, ${x}_{6}=d{\text{r}}/d\xi$, and the control input as ${u}_{1}={\gamma }_{t}$, the state space of the modified model is as follows:

13 $$\dot{x}_{1} = \frac{1}{{4l_{C} B^{2} }}\left[ {x_{2} - u_{1} \sqrt {x_{1} } } \right]$$

14 $$\dot{x}_{2} = x_{3}$$

15 $$\dot{x}_{3} = \mp \frac{Z}{{2\psi^{\prime}_{c} }}\left\{ {\left[ { - \frac{{x_{1} - x_{1C0} }}{H} + 1 + \frac{3}{2}\left( {\frac{{x_{2} }}{W} - 1} \right)\left( {1 - \frac{1}{2}x_{4}^{2} } \right)\, - \frac{1}{2}\left( {\frac{{x_{2} }}{W} - 1} \right)^{3} } \right]H - l_{c} x_{3} } \right\}$$

16 $$\dot{x}_{4} = x_{5}$$

17 $$\dot{x}_{5} = \mp \frac{Z}{{2\psi^{\prime}_{c} }}\left\{ { - \left( {m + \frac{1}{a}} \right)x_{5} - \left( { \pm \frac{{2\psi^{\prime}_{c} }}{Z}x_{6}^{2} \mp \frac{{2\psi^{\prime}_{c} }}{Z}x_{6} \pm \frac{{\psi^{\prime}_{c} }}{Z}} \right)x_{4} \, + \,\frac{3}{2}\frac{{x_{4} H}}{W}\left[ {1 - \left( {\frac{{x_{2} }}{W} - 1} \right)^{2} - \frac{1}{4}x_{4}^{2} } \right]} \right\}$$

18 $$\dot{x}_{6} = \pm \frac{Z}{{2\psi^{\prime}_{c} x_{4} }}\left\{ { - \left( { \pm \frac{{4\psi^{\prime}_{c} }}{Z}x_{5} - mx_{4} - \frac{1}{a}x_{4} } \right)x_{6} - \left( {\frac{1}{2a}x_{4} \mp \frac{{2\psi^{\prime}_{c} }}{Z}x_{5} } \right)} \right\}$$

### Equilibrium point

The equilibrium point is the point at which the rate of change for all state variables is zero. Thus, by setting the differential Eqs. 13–18 equal to zero and solving them for x, the equilibrium points of the compression system are obtained, so the equilibrium region for the Moore-Greitzer equations, according to Eqs. 2–4, is in the following interval (Eq. 19), and the locus of the places where rotating stall stops is provided in Eq. 20.

19 $$\Phi = [0,\,2W]$$

20 $$\Psi = \psi_{c0} + H\left[ {1 - \frac{3}{2}\left( {\frac{\Phi }{W} - 1} \right) + \frac{5}{2}\left( {\frac{\Phi }{W} - 1} \right)^{3} } \right]$$

As can be observed, the interval of changes in the rotating stall flow coefficient and the locus of the points for the start of the rotating stall from various locations are always the same. The equations derived by Shahriyari et al^41^. (Eqs. 5–8), on the other hand, predict the interval of changes in the rotating stall flow coefficient and the location of the instability points based on the slope of the characteristic curve and characteristic curve steepness (W/H) as follows:

21 $$\Phi = [W - W\sqrt {1 - \frac{2}{3}\psi^{\prime}_{c} \frac{W}{H}} ,W + W\sqrt {1 - \frac{2}{3}\psi^{\prime}_{c} \frac{W}{H}} ]$$

22 $$\Psi = \psi_{c0} + H\left[ {1 - \left( {\frac{3}{2} - 2\psi^{\prime}_{c} \frac{W}{H}} \right)\left( {\frac{\Phi }{W} - 1} \right) + \frac{5}{2}\left( {\frac{\Phi }{W} - 1} \right)^{3} } \right]$$

It is worth noting that the Moore-Geitzer (Moore & Greitzer^4^) and Shahriyari et al^41^. Equations are similar when the slope of the characteristic curve is zero.

**Figure 3:**
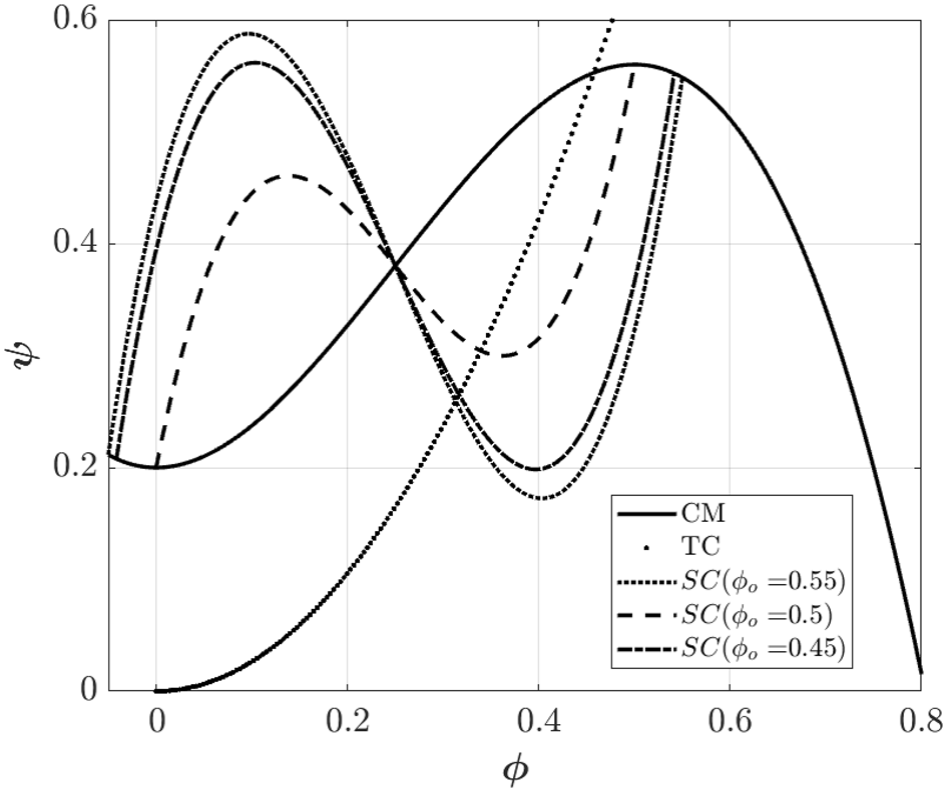
Compressor, stall, and throttle characteristics.

Figure 3 shows the compressor characteristic as well as rotating stall characteristic curves at various initial points (different initial slopes). As can be seen, if the instability can start from a point with a negative slope, as the slope increases, the rotating stall interval equilibrium point also increases. However, if the rotating stall begins in the area between the peak pressure rise and the turning point (note that the slope of the curve is positive), the interval between the rotating stall’s equilibrium points reduces as the slope of the characteristic curve increases.

In a fully-developed rotating stall condition, $d{\text{A}}/d\xi$ is equivalent to zero. Therefore, from Eq. 4 the final amplitude of the stall cell in the MG model becomes (A_e_ is the amplitude of the fully developed stall cell):

23 $$A_{e}^{2} = 4\left[ {1 - \left( {\frac{\Phi }{W} - 1} \right)^{2} } \right]$$

In the model developed by Shahriyari et al^41^., however, the final amplitude of the stall cell is obtained by equating the terms $d{\text{A}}/d\xi$ and $d^{2} A/d\xi^{2}$ in Eq. 7 to zero, which gives the following equation:

24 $$\left( { \pm \frac{{2\psi^{\prime}_{c} }}{Z}\left( {\frac{dr}{{d\xi }}} \right)^{2} \mp \frac{{2\psi^{\prime}_{c} }}{Z}\frac{dr}{{d\xi }} \pm \frac{{\psi^{\prime}_{c} }}{Z}} \right)A = \,\frac{3}{2}\frac{AH}{W}\left[ {1 - \left( {\frac{\Phi }{W} - 1} \right)^{2} - \frac{1}{4}A^{2} } \right]$$

Now using Eq. 25, which is the speed of the stall cell, and Eq. 26 (see Shahriyari et al^41^.), the final amplitude of the stall cell becomes Eq. 27.

25 $$\frac{dr}{{d\xi }} = \frac{1/2}{{1 + am}} = f$$

26 $$\left( {2f^{2} - 2f + 1} \right)A = \,Z$$

27 $$A_{e}^{2} = 4\left[ {1 - \left( {\frac{\Phi }{W} - 1} \right)^{2} } \right] - \frac{8}{3}\psi^{\prime}_{c} \frac{W}{H}$$

**Figure 4:**
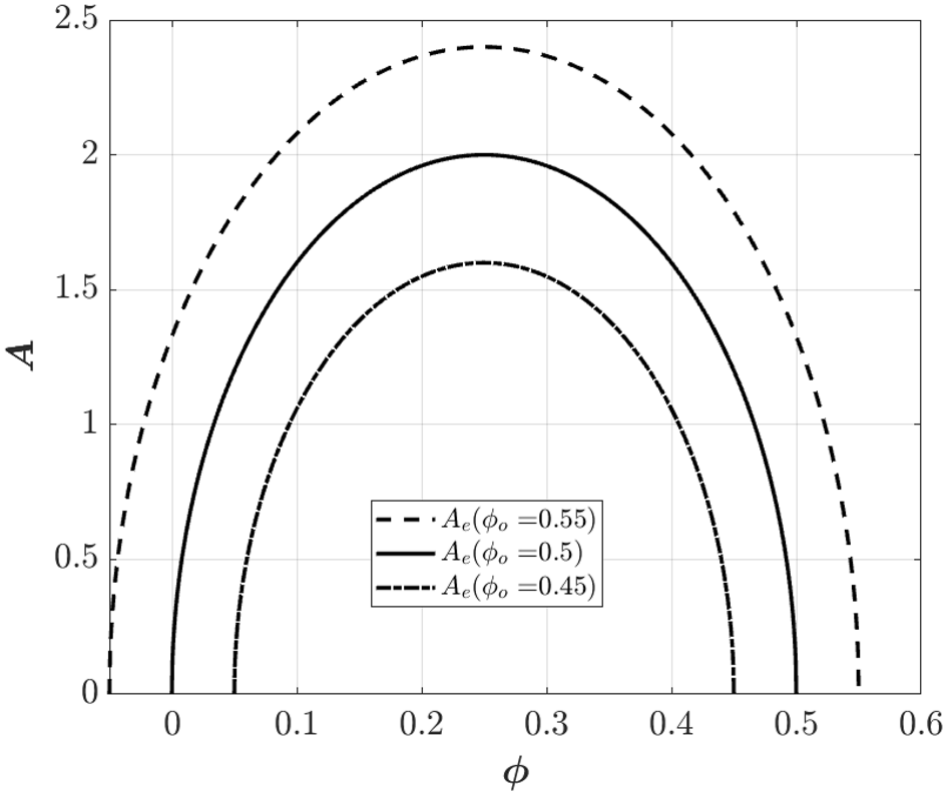
Final amplitude of the stall cell as a function of flow coefficient.

As this equation shows, the final amplitude of the stall cell is dependent on the value of W/H and the slope of the compressor characteristic curve. Figure 4 shows the final stall cell amplitude as a function of the flow coefficient. Φ_0_ is the initial flow coefficient and corresponds to the slope of the compressor characteristic curve. At Φ_0_ equivalent to 0.5, the slope of the characteristic curve is zero. This figure shows that changing the characteristic slope from a negative value (Φ_0_ = 0.55) to a positive one (Φ_0_ = 0.45), decreases the amplitude of the stall cell.

## Pure surge

The model used in this paper is a modified new model. The usual approach to face such a system is to linearize it about the operating points and analyze the local linear behavior of the system inside the domain of attraction of the operating point.

Therefore, the SK model is linearized about the pure surge equilibrium point. The linearized model about the pure surge represents the system’s dynamic behavior in the neighborhood of ${{\varvec{x}}}_{{\varvec{e}}}={\left[{x}_{1e},{x}_{2e}{,x}_{3e }\right]}^{T}$.

28 $$\left[ {\begin{array}{*{20}c} {\dot{x}_{1} } \\ {\dot{x}_{2} } \\ {\dot{x}_{3} } \\ \end{array} } \right] = F_{surge} \left[ {\begin{array}{*{20}c} {x_{1} - x_{1e} } \\ {x_{2} - x_{2e} } \\ {x_{3} - x_{3e} } \\ \end{array} } \right] + G_{surge} \left[ {u_{1} - u_{1e} } \right]$$

with

29 $$\begin{gathered} F_{surge} = \left[ {\begin{array}{*{20}c} { - \frac{1}{{lcB^{2} }}\underbrace {{\frac{{u_{{1_{e} }} }}{{8\sqrt {x_{1e} } }}}}_{{\alpha_{1} }}} & {\frac{1}{{4l_{C} B^{2} }}} & 0 \\ 0 & 0 & 1 \\ { - \underbrace {{\frac{Z}{{ \mp 2\psi^{\prime}_{c} }}}}_{{\psi^{\prime\prime}_{c} }}} & { - \frac{Z}{{ \mp 2\psi^{\prime}_{c} }}\left[ {\underbrace {{\frac{3H}{{2W}}\left( {\frac{1}{2}x_{4e}^{2} - 1} \right)}}_{{\alpha_{2} }} + \underbrace {{\frac{3H}{{2W}}\left( {\frac{{x_{2e} }}{W} - 1} \right)^{2} }}_{{\alpha_{3} }}} \right]} & { - \frac{{Zl_{c} }}{{ \mp 2\psi^{\prime}_{c} }}} \\ \end{array} } \right], \hfill \\ G_{surge} = \left[ {\begin{array}{*{20}c} {\frac{ - 1}{{4l_{C} B^{2} }}\sqrt {x_{1e} } } & 0 & 0 \\ \end{array} } \right]^{T} \hfill \\ \end{gathered}$$

In the neighborhood of pure surge, the local stability of the system is governed by the eigenvalues of the system matrix:

30 $$F_{surge} = \left[ {\begin{array}{*{20}c} { - \frac{1}{{lcB^{2} }}\alpha_{1} } & {\frac{1}{{4l_{C} B^{2} }}} & 0 \\ 0 & 0 & 1 \\ { - \psi^{\prime\prime}_{c} } & { - \psi^{\prime\prime}_{c} \left[ {\alpha_{2} + \alpha_{3} } \right]} & { - \psi^{\prime\prime}_{c} l_{c} } \\ \end{array} } \right]$$

Stability analysis of control systems in the form of their state space representation can be determined by the locus of eigenvalues of the system matrix. In a linearized state space representation, the eigenvalues of the system matrix ($F_{surge}$) correspond to the roots of the $\left| {\lambda I - F_{surge} } \right|$, which is called the characteristic equation. The characteristic polynomial in controllable canonical form is given by:

31 $$\left| {\lambda I - F_{surge} } \right| = s_{0} + s_{1} \lambda + s_{2} \lambda^{2} + s_{3} \lambda^{3} = 0$$

where

32 $$\begin{gathered} s_{0} = \left( {1 + {4}\alpha_{1} \alpha_{2} + {4}\alpha_{1} \alpha_{3} } \right)\psi^{\prime\prime}_{c} \, \hfill \\ s_{1} = \left( {4\alpha_{1} lc + 4l_{c} B^{2} \alpha_{2} + 4l_{c} B^{2} \alpha_{3} } \right)\psi^{\prime\prime}_{c} \hfill \\ s_{2} = 4\alpha_{1} + 4lc^{2} B^{2} \psi^{\prime\prime}_{c} \hfill \\ s_{3} = 4l_{c} B^{2} \hfill \\ \end{gathered}$$

The general stability rule of continuous time linear systems is based on these principles:

- If all the eigenvalues of the system matrix evaluated at the equilibrium point have negative real parts, the system is stable (oscillatory or asymptotic).
- If at least one of the eigenvalues has a positive real part, the system is unstable.

Hopf bifurcation occurs if s_1_ s_2_ = s_0_ s_3_ and s_1_/s_3_ > 0, in which case the eigenvalues are given by ${\lambda }_{\mathrm{1,2}}=\pm i\sqrt{{s}_{1}/{s}_{3}}$ and ${\lambda }_{3}=-{s}_{2}/{s}_{3}$.

Although other bifurcations can be analyzed similarly, it is preferred to obtain the bifurcation diagrams using numerical techniques in Sect. “Bifurcation analysis of MG and SK”.

## Fully-developed rotating stall

For analysing the linear behaviour of the compressor about the fully developed rotating stall, the SK model is linearized about its equilibrium point (${{\varvec{x}}}_{{\varvec{e}}}={\left[{x}_{1e}, {x}_{2e},{x}_{3e }=0, {x}_{4e}, {x}_{5e}{, x}_{6e}=1/3\right]}^{T}$):

33 $$\left[ {\begin{array}{*{20}c} {\begin{array}{*{20}c} {\dot{x}_{1} } \\ {\dot{x}_{2} } \\ {\dot{x}_{3} } \\ \end{array} } \\ {\dot{x}_{4} } \\ {\dot{x}_{5} } \\ {\dot{x}_{6} } \\ \end{array} } \right] = F_{stall} \left[ {\begin{array}{*{20}c} {\begin{array}{*{20}c} {x_{1} - x_{1e} } \\ {x_{2} - x_{2e} } \\ {x_{3} - 0} \\ \end{array} } \\ {x_{4} - x_{4e} } \\ {x_{5} - 0} \\ {x_{6} - \frac{1}{3}} \\ \end{array} } \right] + G_{stall} \left[ {u_{1} - u_{1e} } \right]$$

with

$$F_{stall} = \left[ {\begin{array}{*{20}c} {F_{surge} } & {\left[ {\begin{array}{*{20}c} 0 \\ 0 \\ { - \frac{3}{2}\frac{ZH}{{ \mp 2\psi^{\prime}_{c} }}\left( {\frac{{x_{2e} }}{W} - 1} \right)} \\ \end{array} } \right]} & {{\mathbf{0}}_{3*1} } & {{\mathbf{0}}_{3*1} } \\ {{\mathbf{0}}_{3*3} } & 0 & 1 & 0 \\ {\left[ {\begin{array}{*{20}c} 0 & { - \frac{3Z}{{ \mp 2\psi^{\prime}_{c} }}\frac{{x_{4e} H}}{{W^{2} }}\left( {\frac{{x_{2e} }}{W} - 1} \right)} & 0 \\ \end{array} } \right]} & {F_{54} } & {F_{55} } & {F_{56} } \\ {{\mathbf{0}}_{3*3} } & 0 & {F_{65} } & {\frac{Z}{{ \pm 2\psi^{\prime}_{c} }}\left( {m + \frac{1}{a}} \right)} \\ \end{array} } \right]$$

34 $$\begin{gathered} F_{54} = \frac{Z}{{ \mp 2\psi^{\prime}_{c} }}\left\{ \begin{gathered} \left( { \pm \frac{{2\psi^{\prime}_{c} }}{Z}\left( \frac{1}{9} \right) \mp \frac{{2\psi^{\prime}_{c} }}{Z}\left( \frac{1}{3} \right) \pm \frac{{\psi^{\prime}_{c} }}{Z}} \right) + \hfill \\ \,\frac{3}{2}\frac{H}{W}\left[ {1 - \left( {\frac{{x_{2e} }}{W} - 1} \right)^{2} - \frac{1}{4}x_{4e}^{2} } \right] - \frac{3}{4}\frac{{x_{4e}^{2} H}}{W} \hfill \\ \end{gathered} \right\} \hfill \\ F_{55} = - \frac{Z}{{ \mp 2\psi^{\prime}_{c} }}\left( {m + \frac{1}{a}} \right) \hfill \\ F_{56} = - \frac{{Zx_{4e} }}{{ \mp 2\psi^{\prime}_{c} }}\left( { \pm \frac{{4\psi^{\prime}_{c} }}{Z}\left( \frac{1}{3} \right) \mp \frac{{2\psi^{\prime}_{c} }}{Z}} \right) \hfill \\ F_{65} = \frac{1}{{ \pm 2x_{4e} }}\left\{ { - \left( { \pm 4} \right)\left( \frac{1}{3} \right) - \left( { \mp 2} \right)} \right\} \hfill \\ G_{stall} = \left[ {\begin{array}{*{20}c} {\frac{ - 1}{{4l_{C} B^{2} }}\sqrt {x_{1e} } } & 0 & 0 & 0 & 0 & 0 \\ \end{array} } \right]^{T} \hfill \\ \end{gathered}$$

Each equilibrium point of the linear model is considered stable or unstable according to the principles of stability stated in the previous section.

## Bifurcation analysis of MG and SK

This section examines the effects of throttle gain as a parameter for bifurcation analysis of the modified model using numerical continuation techniques. Numerical continuation techniques track the equilibrium points of the compression system and their global bifurcations in the range of throttle gain variations.

**Figure 5:**
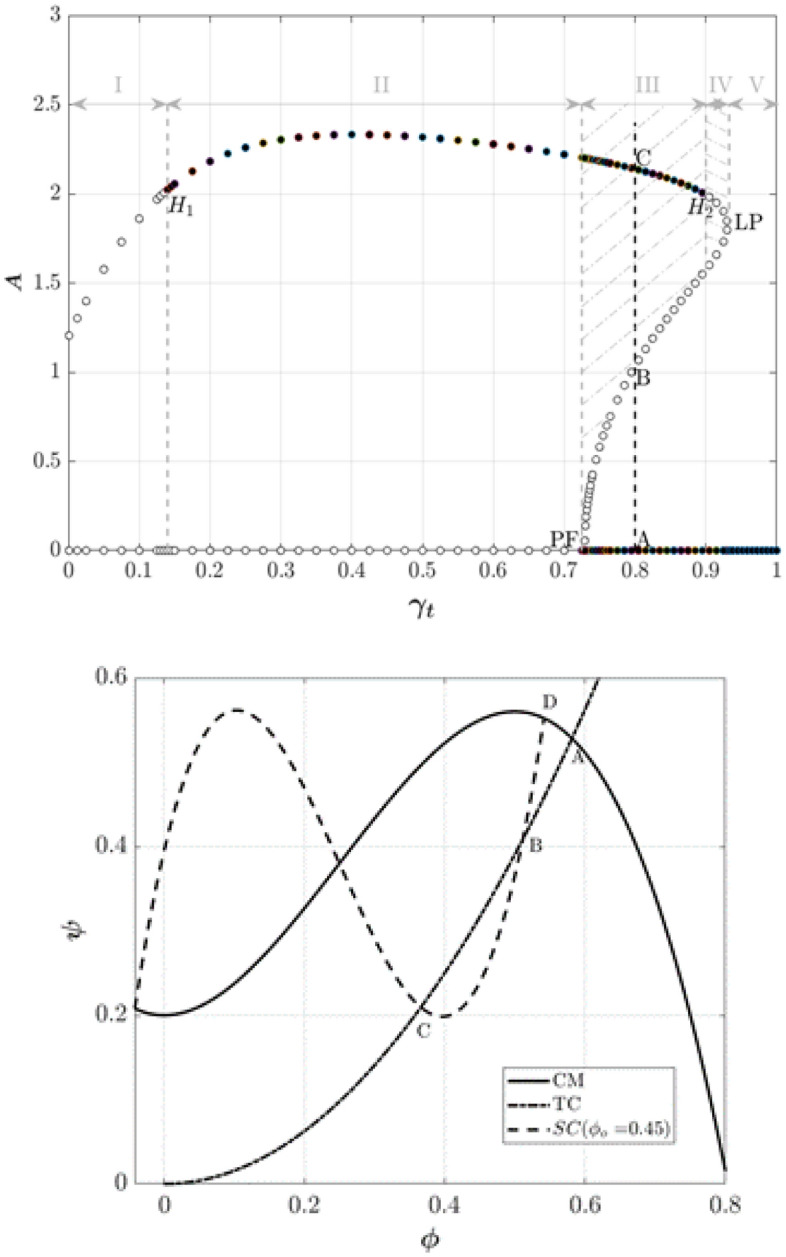
Final amplitude of the stall cell versus the position of the throttle valve (stable point: solid circles, unstable points: hallow circles).

Figure 5 shows the bifurcation diagram of the final amplitude of the stall cell (A) versus the position of the throttle valve (${\gamma }_{t}$). In this figure, all trajectories starting from an initial point in regions I and IV converge to a limit cycle, representing a surge cycle. On the other hand, all trajectories starting from an initial condition in regions II and V converge to a fully developed rotational stall and a fully damped rotating stall, respectively. In Region III, the compression system might experience a surge or rotating stall.

The bifurcation point (PF) is a subcritical pitchfork bifurcation associated with the hysteresis loop in the rotating stall. At this point, the throttle characteristic intersects the compressor map and stall characteristic at two points, one of which is the operating point (D). The limit point (LP) represents a throttle gain where the throttle characteristic is tangent to the stall characteristic. For all throttle gains greater than LP, the compressor system, regardless of the initial perturbation value, ends up at a fully damped rotating stall, which represents the stable operating points of the compressor. Stall may be formed or damped out by decreasing the throttle gain to a value between LP and PF, depending on its initial value. In this case, operating point (C) is a fully developed rotational stall, operating point (A) is a stable operating point and operating point (B) is an unstable operating point. The first Hopf bifurcation point (H1) and the second Hopf bifurcation point (H2) promise the inception of surge (see for more details^21^). Figure 5 also exhibits hysteresis. Assume that the system has entered a fully developed stall condition at operating point (C). The stall may be removed by opening the throttle. The mass flow rate increases, but the system cannot return to the stable point until the throttle characteristic is tangent to the stall characteristic (LP), where the operating point jumps to a completely damped stall corresponding to the operating point (A).

**Figure 6:**
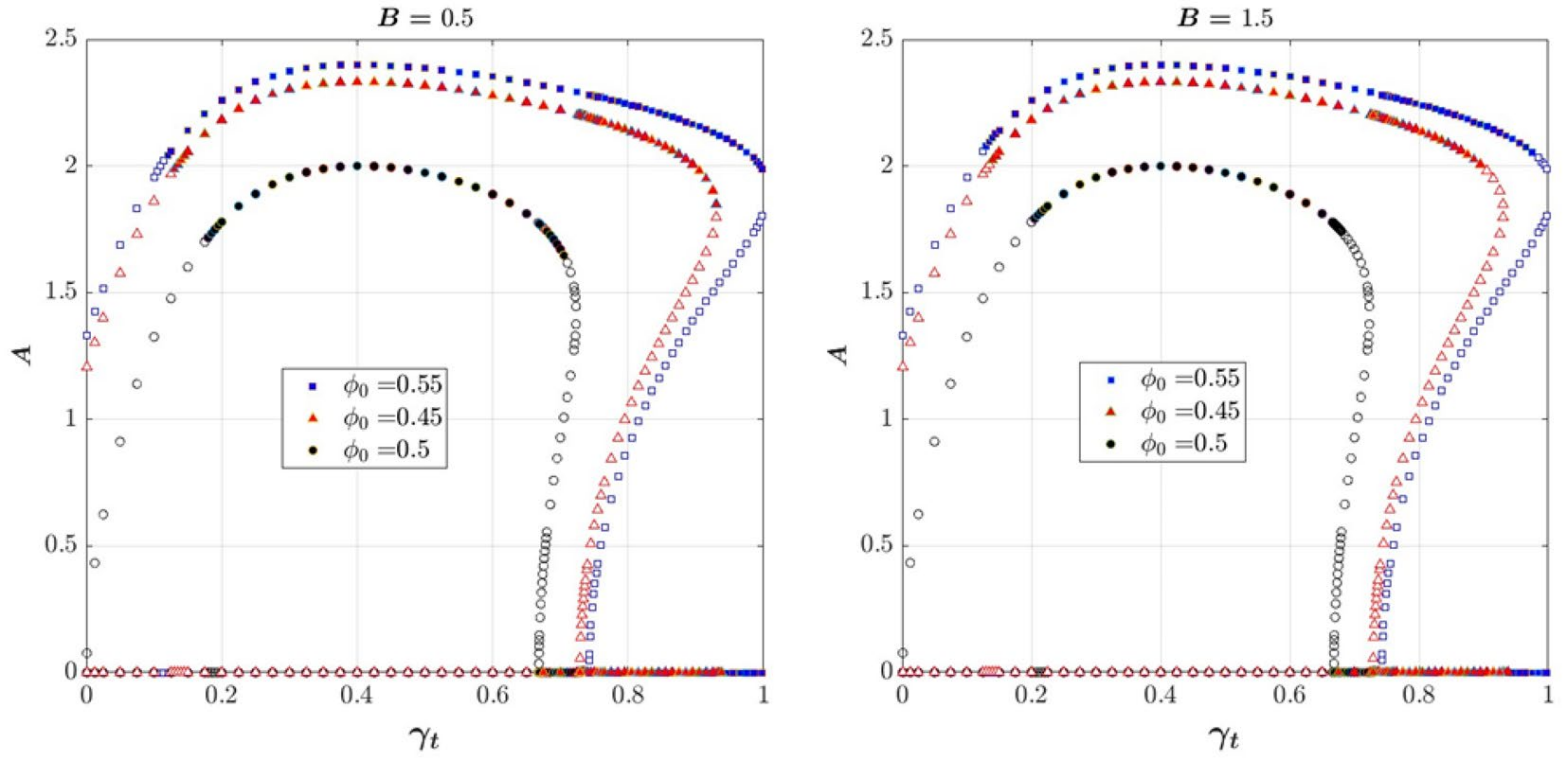
Two-dimensional bifurcation diagram: solids show the stable and hollows show the unstable equilibrium points.

Figure 6 shows the bifurcation diagram for three initial operating points (${\Phi }_{0}$= 0.45, 0.5 and 0.55) and two Greitzer parameters, B = 0.5, 1.5. Note that the diagram corresponding to ${\Phi }_{0}$= 0.5 is the same as that of MG, which does not change with the initial flow coefficient.

Comparing Figs. 5 and 6 shows that Bifurcation points H2 and region IV which were described above, appear in the bifurcation diagrams only for higher values of B (e.g. B = 1.5). When the B-parameter increases to a higher value (e.g. B = 1.5), or the initial operating point moves away from the peak pressure rise point (e.g.${\Phi }_{0}$ = 0.45 and 0.55), the range of regions I and III increases and the range of regions II and IV decreases.

## Control of surge and rotating stall

This section delves into the stability and active control of axial compression systems, focusing on the nonlinear phenomena of surge and rotating stall. Linearizing the nonlinear model around operating points is advantageous for analysis and control. This enables the investigation of the system’s linear behavior within each operating point's attraction domains. Due to its ease of design, stability guarantees, and optimality, the LQR controller was chosen to stabilize the compressor effectively and suppress rotating stall and surge limit cycles through throttle valve actuation (Fig. 1 illustrates the position of the sensors and throttle valve actuation as control input signals).

It is crucial to note that the LQR controller, despite being linear, was validated using the nonlinear dynamic model. This section evaluates the impact of nonlinearities on the performance of the LQR controller.

Considering the linear model of the compression system (Eq. 35), the LQR control design aims to compute the optimal feedback gain (matrix **K)** such that the feedback control law (Eq. 36) minimizes a quadratic cost function (Eq. 37).

35 $${\dot{\mathbf{x}}} = {\mathbf{F}}_{surge/stall} {\mathbf{x}} + {\mathbf{G}}_{surg/stall} {\mathbf{u}}$$

36 $${\mathbf{u}} = {\mathbf{Kx}}$$

37 $$J = \int\limits_{0}^{t} {\left( {{\mathbf{x}}^{T} {\mathbf{Qx}} + {\mathbf{u}}^{T} {\mathbf{Ru}}} \right)\,dt}$$

The tuning matrices **Q** and **R**, respectively representing the state and control penalty matrices, play a crucial role in striking a balance between state tracking accuracy and control effort. Increasing the value of **Q** expedites the convergence of state vector errors, while a similar increment in **R** leads to reduced control efforts. Striking a balance between these two parameters is essential for achieving desirable performance. Whenever the linear model is controllable, the LQR method effectively stabilizes the unstable system by ensuring that all eigenvalues of the system possess negative real parts.

The closed loop system is:

38 $${\dot{\mathbf{x}}} = \left( {{\mathbf{F}}_{surge/stall} + {\mathbf{G}}_{surg/stall} {\mathbf{K}}} \right){\mathbf{x}},\quad x(0) = x_{0}$$

And the closed loop cost is expressed by:

39 $$J = \int\limits_{0}^{t} {{\mathbf{x}}^{T} \left( {{\mathbf{Q}} + {\mathbf{K}}^{T} {\mathbf{RK}}} \right){\mathbf{x}}\,dt} = x_{0}^{T} \underbrace {{\left( {\int\limits_{0}^{t} {e^{{\left( {{\mathbf{F}}_{surge/stall} + {\mathbf{G}}_{surg/stall} {\mathbf{K}}} \right)^{T} t}} \left( {{\mathbf{Q}} + {\mathbf{K}}^{T} {\mathbf{RK}}} \right)e^{{\left( {{\mathbf{F}}_{surge/stall} + {\mathbf{G}}_{surg/stall} {\mathbf{K}}} \right)t}} \,dt} } \right)}}_{{\mathbf{P}}}x_{0}$$

Let the optimal u^*^ be expressed in terms of **P**

40 $${\mathbf{u}}^{*} = {\mathbf{Kx}} = \left( { - {\mathbf{R}}^{ - 1} {\mathbf{G}}_{surg/stall} {\mathbf{P}}} \right){\mathbf{x}}$$

Then **P** can be solved backward in time (Eq. 41, which is called Riccati algebraic Equation^42^.

41 $$0 = {\mathbf{PF}}_{surge/stall} + {\mathbf{F}}_{surge/stall}^{T} {\mathbf{P}} - {\mathbf{PG}}_{surg/stall} {\mathbf{R}}^{ - 1} {\mathbf{G}}_{surg/stall}^{T} {\mathbf{P}} + {\mathbf{Q}}$$

In an optimal controller, the throttle valve should be able to quickly reach the desired point by detecting the growth of disturbances. To achieve this goal, it is necessary to select the control coefficients so that the throttle coefficient is greater than the compressor flow coefficient and the compressor flow coefficient is greater than the pressure increase coefficient. Therefore, the state and control penalty matrices are set as Q = diag([1, 10, 1]), R = 100.

The operating point (OP) of the compression system is the intersection of the throttle characteristic and the equivalent compressor map. The task defined for the LQR controller is to bring the compression system states to the vicinity of the peak pressure rise point, so that the compressor can operate stably at the highest performance (with no disturbance in the compression system). The desired point is located at the negative slope region (Φ_d_ = 0.51), which is inherently stable. Table 2 indicates the Greitzer parameter, a sign of characteristic slop of the initial operating point, and initial disturbance amplitude, for six different simulated scenarios.

**Table 2:** Simulated scenarios.

Scenario number	Greitzer parameter (B)	Sign of char. Slops (initial operating point)	Initial disturbance amp. (A(0))
1	0.5 (expecting rotating stall)	Positive	0.01
2	0.5 (expecting rotating stall)	Positive	0.2
3	0.5 (expecting rotating stall)	Negative	0.01
4	0.5 (expecting rotating stall)	Negative	0.4
5	1.5 (expecting surge)	Positive	0.01

**Figure 7:**
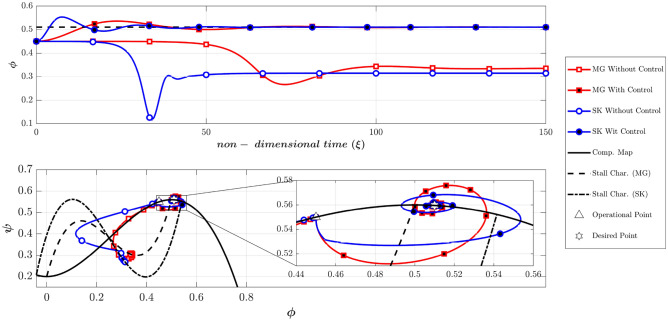
Dynamic behavior of the compression system with and without LQR controller (scenario 1).

**Figure 8:**
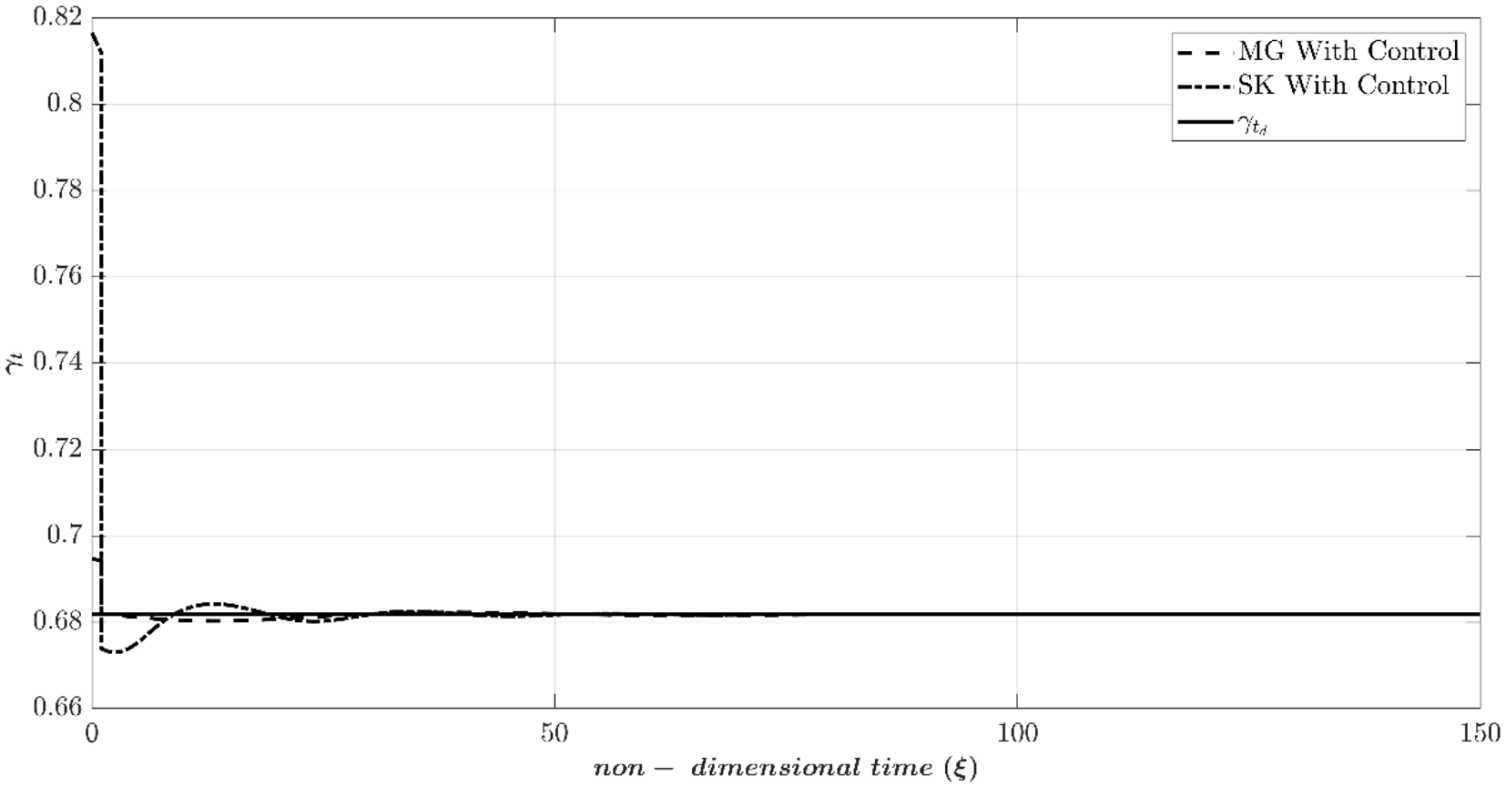
Time history of throttle gain (scenario 1).

According to Table 2, Fig. 7 compares the dynamic behavior of the Moore-Greitzer (MG) model and the modified model (SK) with and without the controller in scenario one. The stall characteristic curves for MG and SK models are shown in this figure. Without the controller, the system starts from OP (the intersection of the throttle characteristic γ_t_ = 0.6068 and the compressor map at (Φ_0_, Ψ_0_) = (0.45, 0.55), which is in the positive slope region. At this point, the initial amplitude of perturbation (A_0_) is equivalent to 0.01. As illustrated in this figure, both models lead to stall (i.e., in both models the flow coefficient reduces and converges to a fully developed stall point on its corresponding stall characteristic). However, the LQR controller effectively stabilizes the rotating stall and brings both systems similarly to the desired point (OP_d_ = 0.51). Figure 8 shows the efforts of LQR control (the throttle gain), for MG and SK models. The desired value of the throttle gain is plotted in this figure by solid line. As demonstrated, the control effort of both models can rapidly and satisfactorily stabilize the compressor to the desired point (Φ_d_ = 0.51 in Fig. 7).

**Figure 9:**
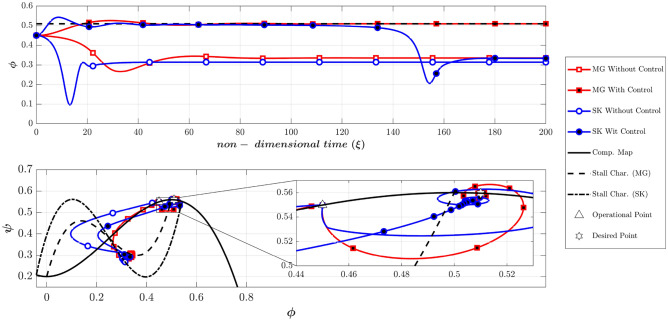
Dynamic behavior of the compression system with and without LQR controller (scenario 2).

In scenario two (as presented in Table 2), the effects of the initial perturbation amplitude on the performance of the open-loop and closed-loop systems are studied by significantly increasing A_0_ to 0.2. Figure 9 provides a comparison of the dynamic behavior of the compressor, similar to Fig. 7. This figure reveals that the uncontrolled system becomes unstable in both models.

The Moore-Greitzer model demonstrates that despite the high disturbance of A(0) = 0.2, the controller can successfully stabilize the compressor and converge to the desired point. Conversely, in the SK model, although the controller initially attempts to stabilize the system at the desired point, it ultimately diverges and converges to another point with a lower flow coefficient.

As will be explained, this suggests that the SK model is more realistic in this example because A(0) = 0.2 is a very intense disturbance which could not be controlled. In order to justify this claim, it should be stated that in SK model (also in MG^43^), the disturbance term in the flow coefficient is defined based on the following equation:

42 $$\phi = \Phi \left( \xi \right) + WA\left( \xi \right)\sin \left( {\theta - f\xi } \right)$$

The value of the *WA(0)* shows the amplitude of the initial disturbances (it should be noted that the value of W is constant and is equal to 0.25 in this study and only the value of A(0) is variable as the initial value). Therefore, with the initial flow coefficient of 0.5 and the initial A(0) = 0.001, 0.01 and 0.1 the amplitude of disturbances will be 0.1%, 1% and 10%, respectively. As stated by Greitzer and Moore^39^, disturbances with an initial amplitude of 0.1% correspond to a disturbance of a good wind-tunnel test section, the 1% level is a reasonable estimate for the magnitude of disturbances prior to rotating stall. However, the 10% level is far beyond the range of usual disturbances before instability occurs. It is expected that the linear controller can control the initial disturbances with the amplitude of 0.1% and 1%, but cannot control the disturbances with the level of 10%^39^. In the above scenario, the value of A(0) was equivalent to 0.2 (equivalent to 20% amplitude of disturbance), but the MG model controlled the disturbances, which cannot occur in a real application.

**Figure 10:**
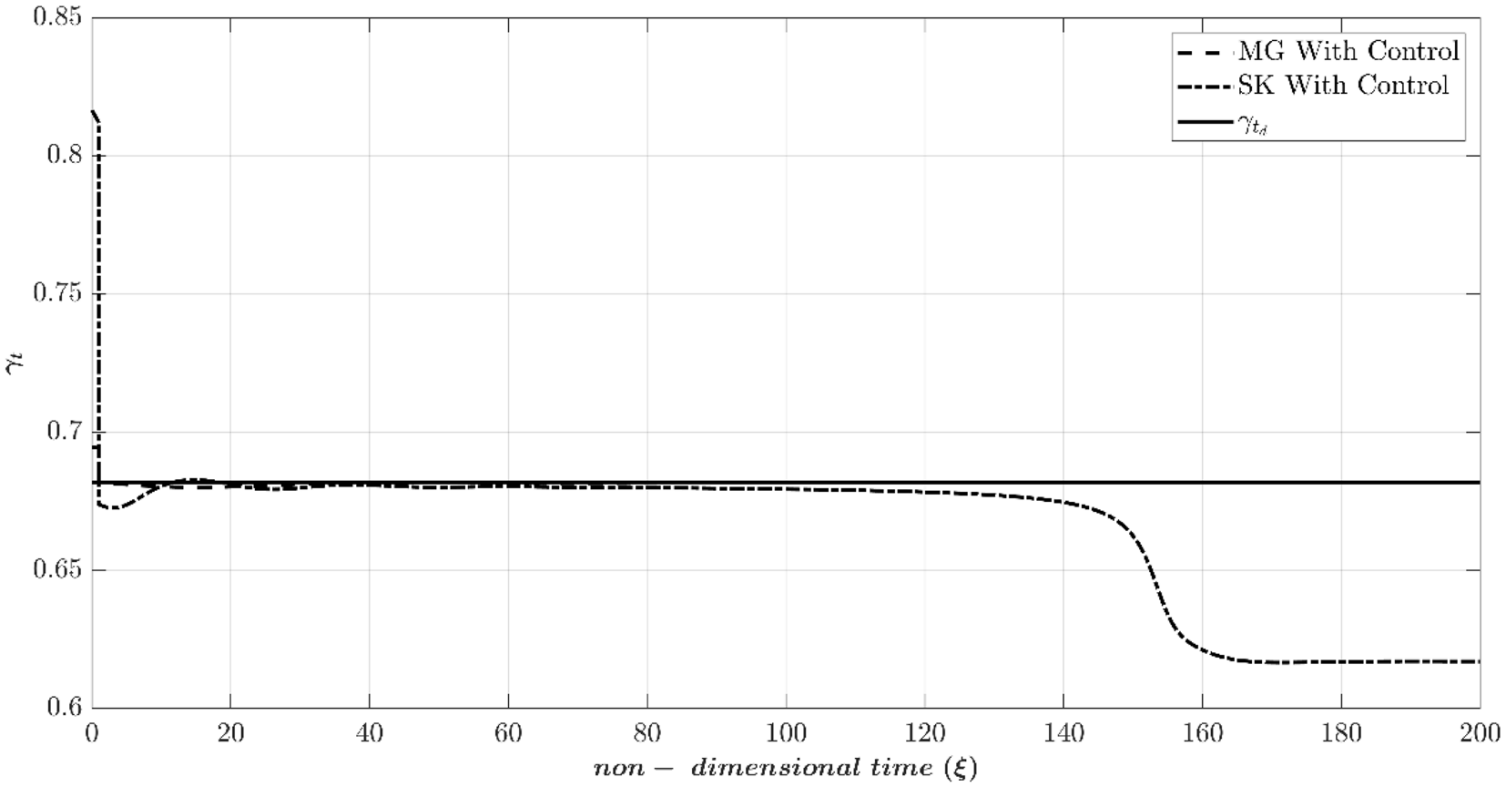
Time history of throttle gain (scenario 2).

The reason is that (despite the MG model) the SK model can simulate the instability initiation when the initial operating condition is located in the negative compressor characteristic slope^41^. Because the initial disturbance is very high, the nonlinear system deviates from the attraction basin of the linear LQR controller. As a result, although the controller attempts to converge the system to the desired point, the nonlinear dynamic behavior of the system leads it to the equilibrium point of another attraction basin. This highlights the importance of the initial condition not being far from the desired point in linear controllers, as the system may end up in the attraction basin of another operating point. This phenomenon is not observed in the MG model, which suggests that the SK model can describe the compressor’s nonlinear behavior more accurately. Figure 10 also illustrates this unstable behavior by showing the control efforts similar, to Fig. 8, confirming the above discussion.

It should be noted that the final operating points in the SK model with and without a controller are slightly different (see Fig. 9 top plot), which is due to the difference between the initial and desired throttle gains.

**Figure 11:**
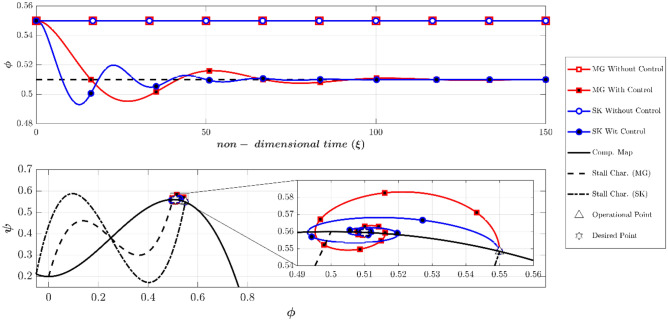
Dynamic behavior of the compression system with and without LQR controller (scenario 3).

According to the Camp and Day^8^, the instability can be initiated from the negative slope region of the compressor. To compare the MG and SK models at such conditions, the dynamic behavior of the two models with and without controllers are depicted in Fig. 11, similar to Fig. 7. Note that the Greitzer parameter (B) is chosen to be 0.5 (scenario 3 according to Table 2), expecting the occurrence of rotating stall. The initial operating (OP) is at (Φ_0_, Ψ_0_) = (0.55, 0.5485). Furthermore, the initial throttle gain which is obtained by intersecting the throttle characteristic and the compressor map at OP, is equivalent to γ_t_ = 0.7426. Also, the initial amplitude of disturbances is chosen to be 0.01 (A(0) = 0.01). As shown in Fig. 11, with no control both models damp the low amplitude disturbances and remain stable. This was expected because the initial operating point was chosen in the stable region of the compressor. With the controller, both of the models have effectively reached the desired point Φ_d_ = 0.51.

**Figure 12:**
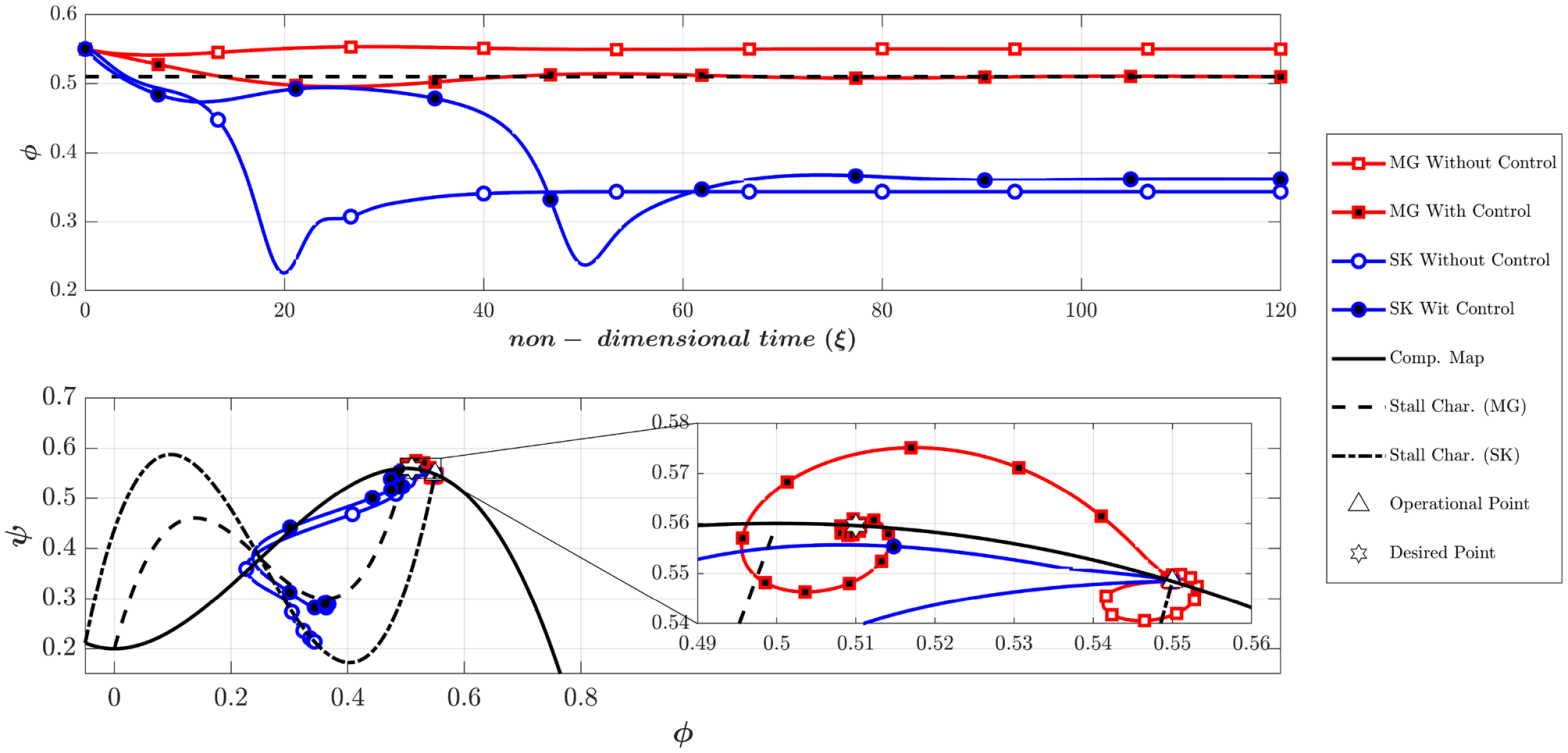
Dynamic behavior of the compression system with and without LQR controller (scenario 4).

To investigate the possibility of the occurrence of a rotating stall from the negative slope region of the compressor, the initial amplitude of the disturbances was chosen to 0.4, which is dramatically high (scenario 4). If the controllers are deactivated (Fig. 12), the MG model spuriously remains stable, but the SK model predicts a fully developed rotating stall. Furthermore, while the controller stabilizes the MG model (even at a high level of disturbances), it cannot prevent the instability in the SK model (Fig. 12). As previously explained, because of the difference between the initial and desired throttle gains, the final operating points in the SK model with and without controller are a bit different (see Fig. 12 top plot).

**Figure 13:**
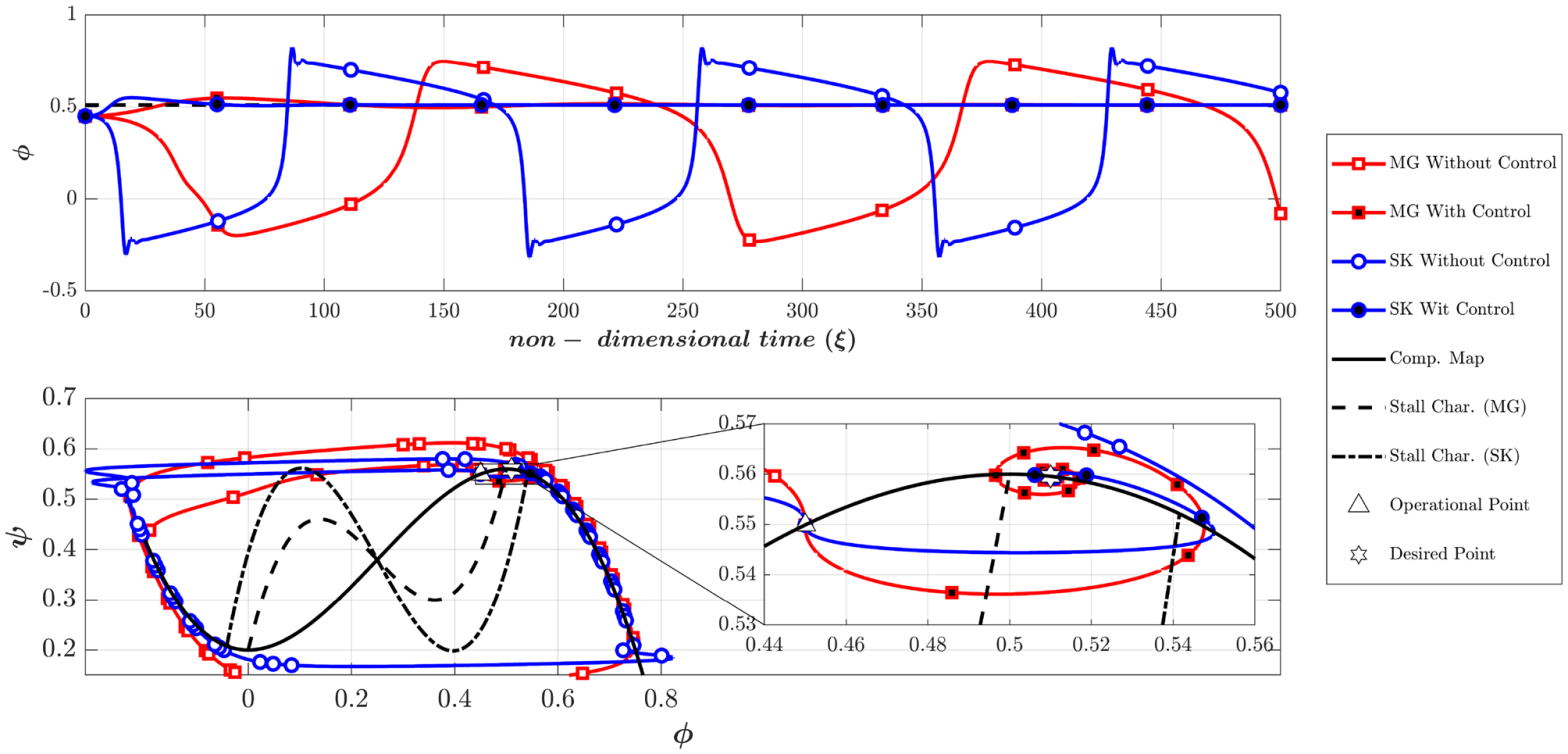
Dynamic behavior of the compression system with and without LQR controller (scenario 5).

The Greitzer parameter (B) is shown to be a key parameter for determining whether the compressor exhibits a surge or rotating stall (4,39). To compare the two models in predicting surge phenomena, the Greitzer parameter has been increased to 1.5 (scenario 5). The initial operating point is similar to scenario 1 and is equal to (Φ_0_, Ψ_0_) = (0.45, 0.55) and the initial amplitude of the disturbances is chosen to be 0.01. As Fig. 13 shows, the dynamic behavior of these two models is similar during surge, when the controller is deactivated. Furthermore, the developed controllers can stabilize the deep surge in the two models.

It should be noted that if the operating point is in the positive slope region, the MG model with the controller, stabilizes the compression system having a surge, even with a very high initial amplitude of the disturbances (similar to the rotating stall discussed in scenario 2). Furthermore, similar to scenarios 3 and 4 if the initial operating point is located in the negative slope region MG model always damped the disturbances regardless of the initial amplitude of the disturbances (with or without a controller). However, it seems that the SK model can give a more realistic prediction at such conditions (i.e., in the SK model the control effort might be successful or unsuccessful, depending on the initial amplitude of disturbances).

## Conclusion

This paper compared the bifurcations and LQR controller for two compressor post-stall models, namely MG and SK. First, MG and SK perturbed linear state-space models about equilibrium points, pure surge, and rotating stalls were developed. Also, the unstable part of the compression system was identified by eigenvalue and global bifurcation analysis. In addition, the closed-loop performances of these models (with optimal model-based LQR controllers) were investigated. The following conclusions can be drawn from this work:

When the amplitude of the disturbance is small and the initial operating point is located at the positive slope portion of the compressor characteristic, the two models lead to instability without a controller. Furthermore, when the LQR controller is activated, it can perfectly stabilize both models.

With an intensive disturbance and a positive slope initial point, the two models predict instability without a controller. With the controller, however, the models behave differently. While the SK model damps a reasonable range of disturbances and predicts instability for very intensive disturbances, the MG model always damps the disturbances even when extremely intense.

In the compressor negative slope region, initial disturbances (even very intense) can never grow and are always damped when there is no controller in the MG model (obviously the same happens with the controller). In the SK model, however, the disturbances might be damped or grown, depending on the amplitude of the disturbances (without a controller). With the controller, if the disturbances are small, the SK model can effectively control the instabilities. Nonetheless, for very intense disturbances the controller fails to dampen the instabilities which is in line with reality.

The most important issues that are not foreseen in the proposed model are the analysis of the sensitivity of the modified model against uncertainties, the robustness of the control model and the design of a robust nonlinear controller for the modified compressor model. We will address these issues in our future work.

## Data Availability

All data generated or analyzed during this study are included in this published article.

## List of symbols

*A*: The amplitude of the first harmonic disturbance
*A*_*e*_: The amplitude of fully developed stall cell
*a*: Time-lag parameter
*B*: Greitzer parameter
*f*: The final speed of disturbances relative to the speed of the blade
*H*: Semi-height of cubic axisymmetric characteristic
*l*_*c*_: Equivalent compressor length
*m*: Outside compressor lag parameter
*r*: Phase angle
*R*: Mean wheel radius
*t*: Time
*U*: Wheel speed at mean diameter
*W*: Semi-width of cubic characteristic
*η*: Axial disturbances measured in wheel radii
*θ*: Angular coordinate around the wheel
*γ*_*T*_: Throttle coefficient
*ξ*: Nondimensional time
Φ: Axial flow coefficient in fan, annulus averaged; axial velocity divided by wheel speed
Φ_*T*_: Flow coefficient of throttle duct
*ψ*: Total-to-static pressure rise coefficient
*ψ*_*c*_: Axisymmetric pressure rise coefficient
*ψ′*_*c*_: Derivative of axisymmetric pressure rise coefficient
*ψ*_*c0*_: Shut-off value of the axisymmetric characteristic
